# Quality of Life for Patients Receiving Elective Interventions for Abdominal Aortic Aneurysms

**DOI:** 10.3390/jpm12060910

**Published:** 2022-05-31

**Authors:** Silvestra Barrena-Blázquez, Manuel Díez-Alonso, Luis Felipe Riera del Moral, Salvador Sanchez Coll, Natalio García-Honduvilla, Melchor Alvarez-Mon, Miguel A. Ortega, Fernando Ruiz-Grande

**Affiliations:** 1 Department of General Surgery, Príncipe de Asturias Hospital, 28801 Alcalá de Henares, Spain; manuelmariano.diez@salud.madrid.org; 2 Department of Surgery, Medical and Social Sciences, Faculty of Medicine and Health Sciences, University of Alcalá, 28801 Alcalá de Henares, Spain; 3 Department of Vascular Surgery, Nuestra Señora del Rosario Hospital, 28834 Madrid, Spain; luis.riera@salud.madrid.org (L.F.R.d.M.); ssanchezc@telefonica.net (S.S.C.); fruizgrande@hotmail.com (F.R.-G.); 4 Department of Medicine and Medical Specialities, Faculty of Medicine and Health Sciences, University of Alcalá, 28801 Alcalá de Henares, Spain; natalio.garcia@uah.es (N.G.-H.); mademons@gmail.com (M.A.-M.); miguel.angel.ortega92@gmail.com (M.A.O.); 5 Ramón y Cajal Institute of Sanitary Research (IRYCIS), 28034 Madrid, Spain; 6 Immune System Diseases-Rheumatology and Internal Medicine Service, University Hospital Príncipe de Asturias, CIBEREHD, 28806 Alcalá de Henares, Spain; 7 Department of Vascular Surgery, Príncesa Hospital, 28834 Madrid, Spain

**Keywords:** abdominal aortic aneurysm, health-related quality of life, open abdominal repair, EVAR, SF-36

## Abstract

**Objectives:** Information on the quality of life of patients operated on for abdominal aortic aneurysm (AAA) is scarce. The objective of this study was to analyse these patients’ health-related quality of life (HRQoL). **Materials and Methods:** This was a cross-sectional observational study. Patients undergoing elective AAA surgery from January 2013 to December 2020 were included. The Spanish version of the SF-36 questionnaire was administered to participants one to sixty months after surgery. **Results:** During the study period, 178 patients underwent surgery for AAA, 109 (61.23%) had open abdominal aortic repair (AAR) and 69 (38.54%) had endovascular aneurysm repair (EVAR). Mortality before the month of surgery was higher among those treated by AAR than EVAR (2.7% and 1.45%, respectively), while late mortality was higher in the EVAR group than in the AAR group (11.5% and 2.7%, respectively). In the late postoperative period, 12.5% of patients who underwent AAR presented complications compared to 25% of those treated with EVAR. The questionnaire was administered to 151 patients (91 AAR and 60 EVAR patients). The AAR patients compared to the EVAR patients had significantly higher mean scores on the health scales of the SF-36 questionnaire in Physical Function (*p* = 0.001), Vitality (*p* = 0.003), General Health (*p* = 0.37), Social Function (*p* = 0.023) and Mental Health (*p* = 0.006). Scores on the Mental Summary Component were significantly higher in the AAR group (*p* = 0.026). **Conclusions:** The group of patients treated with AAR showed the highest average scores on the scales of the SF-36 questionnaire in Physical Function, Vitality, General Health and Mental Health. The worst result was found in the Social Function scale for EVAR patients and was related to a higher rate of late complications.

## 1. Introduction

Abdominal aortic aneurysm (AAA) is a vascular degenerative condition that has a higher incidence in men older than 65 years with a history of smoking. It runs silently until the onset of complications. The main complication is rupture, and it is associated with a very high mortality rate [1]. Depending on the diameter and characteristics of the aneurysm, management options for asymptomatic patients include observation with follow-up, medical therapy, open surgery or placement of covered stents [2]. Elective surgery is recommended when the aneurysm is >5.5 cm, shows progressive growth and produces symptoms or ruptures [3].

Since the introduction of endovascular aneurysm repair (EVAR) at the end of the 1990s as a therapeutic alternative to open abdominal aortic repair (AAR), many clinical studies have been published to compare the results of both treatments. A higher early mortality has been recorded among patients treated with AAR, while a higher late mortality has been reported among patients treated with EVAR due to a greater need for reoperations and late complications [4,5].

The evaluation of health-related quality of life (HRQoL) can be used to measure the results related to the benefits obtained with the different therapeutic methods [6]. However, few studies address the quality of life of these patients or analyse the impact of these therapeutic alternatives. The objective of this study was to describe the HRQoL results of patients with AAA who underwent elective intervention with AAR or EVAR.

## 2. Materials and Methods

This was a cross-sectional observational study. Patients with a diagnosis of infrarenal AAA who underwent AAR or EVAR electively, between January 2013 and December 2020 at the Prince of Asturias University Hospital in Alcalá de Henares and the Nuestra Señora del Rosario Hospital in Madrid, were included. Patients with ruptured, adrenal or inflammatory aneurysms and patients who did not understand the Spanish language were excluded from the analysis. The selection of study participants was performed by reviewing the clinical histories of patients operated on in the Vascular Surgery Unit throughout the study period. The study was developed in accordance with the principles of the Declaration of Helsinki and was approved by the Ethics Committee of the Prince of Asturias Hospital. The patients were informed of the study, and those who agreed to participate signed an informed consent document.

The protocol followed was recommended surgery when the aneurysm had a transverse diameter greater than 5.5 cm in the image of the thoracic-abdominal-pelvic CT with contrast, when the growth of the AAA was greater than 0.5 cm/year, when it produced symptoms, or when it ruptured (extreme emergency surgery). Endoprosthesis was indicated for patients older than 65 years, with high surgical risk and favourable aneurysm morphology (distance to the exit of the renal artery greater than 1.5 cm and absence of angled shapes that would hinder anchorage of the prostheses). The AAR surgical technique included replacements of the diseased aortic section with a prosthesis (Dacron or polytetrafluoroethylene (PTFE)). The EVAR technique was carried out by means of sheaths introduced through the femoral arteries, which allowed the advancement of rigid metal guides that crossed the aneurysm and allowed the deployment of a stent to form a new channel for blood flow, isolating it between two healthy parts of the artery.

The clinical and demographic data of the patients were recorded in a data log prepared specifically for the study and entered into a computerised database with the statistical program Microsoft Excel 2019 (v.19)^R^; access was restricted to the patients and members of the research team. During the entire study, the anonymity of the patients was maintained. Data were collected on comorbidities, cardiovascular risk factors, aneurysm morphology, type of intervention, early postoperative complications (within a month of surgery), late complications (more than a month after surgery), mortality and length of hospital stay. Respiratory distress was defined as an acute, diffuse, inflammatory, non-infectious pulmonary process; associated with increased vascular pulmonary permeability; and characterised by hypoxemia, decreased pulmonary compliance and increased intrapulmonary shunt [7]. Acute renal failure was defined as serum creatinine elevation ≥3 mg/dL or glomerular filtration decrease <75% [8]. Postoperative ileus was defined as insufficiency of the propulsive intestinal motility persisting more lasting than 5 days after surgery. Bronchopneumonia was defined as an inflammatory infectious process affecting diffusely alveolar space and distal bronchial tree.

The measurement instrument used was the SF-36 health questionnaire. This questionnaire consists of 36 questions that encompass 8 dimensions or scales of health: Physical Function (10 items), Physical Role (four items), Body Pain (two items), Vitality (four items), Social Function (two items), Emotional Role (three items) and Mental Health (5 items). In addition, it includes the item “Health Transition”, which asks about the perceived change in the general health status with respect to the previous year. To measure patient responses to the SF-36 questions, the scores for each item are coded, aggregated and transformed into a scale from 0 to 100, where 0 is the worst and 100 is the best health status, using the algorithms, scoring and interpretation in the questionnaire’s manual [9]. The questionnaire allows the calculation of two summary scores: the Physical Summary Component (PCS) (including the scores for Physical Function, Physical Role, Body Pain and General Health) and the Mental Summary Component (MCS) (including the scores for Vitality, Social Function, Emotional Role and Mental Health).

All patients were contacted by telephone to inform them of the study and invite them to participate, and all agreed to collaborate. The majority (89%) went to the hospital, where they were interviewed by the main researcher, who explained the objective of the study in detail, provided written information and obtained informed consent for participation. Patients who could not go to the hospital (11%) were interviewed by telephone. All patients underwent a single interview a month or more after the surgery, recording the time elapsed since the intervention. All interviews were conducted between December 2019 and December 2020.

For each health area included in the SF-36 questionnaire, the mean, median, percentiles (25, 50 and 75), standard deviation and proportion of individuals with the maximum and minimum score (ceiling and floor effect, respectively) were calculated. In addition, the psychometric properties of the health scales of the questionnaire were studied: proportion of nonresponses, reliability through the Cronbach’s alpha coefficient and the correlation of the items that make up each scale with its total score (Spearman’s correlation).

Regarding the variables related to clinical aspects, the categorical variables were expressed by the number of observations and percentages, and we compared the results using the chi-square test. For the continuous variables, the normality of their distribution was analysed (Kolmogorov test), and the results were described by their mean, median, standard deviation and percentiles. Kruskal–Wallis and Mann–Whitney U tests were used to compare the results. For statistical analyses, the SPSS program (v.23) (IBM, Armonk, New York, NY, USA) was used.

## 3. Results

**Figure 1:**
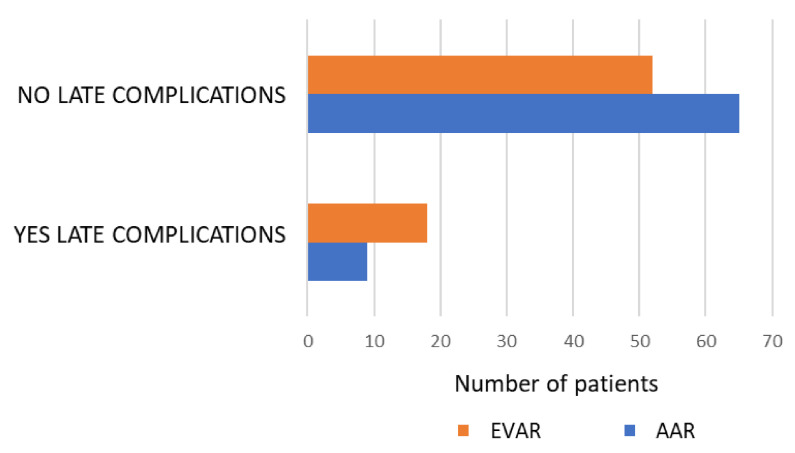
Late complications of AAA due to AAR vs. EVAR. Chi-square comparison: *p* = 0.013.

During the time period studied, 178 patients underwent surgery for AAA, of which 93.8% were men and 6.2% were women. The mean age was 73.54 ± 7.6 years. The median follow-up was 30 months. The difference in age between the patients who underwent AAR and EVAR was not statistically significant (*p* = 0.09). The cardiovascular risk factors found in the patients were arterial hypertension (69.8%), dyslipidaemia (50%), smoking (58%) and associated aneurysms (58.7%). Mortality the month after surgery for patients treated electively for AAA with AAR was 2.7%, and it was 1.45% for patients treated with EVAR. Late mortality for the AAR group was 2.7% and 11.5% for the EVAR group. The general characteristics and postoperative complications of the patients are shown in Table 1. Late postoperative complications were more frequent in the EVAR group (*p* = 0.013) (Figure 1). In the EVAR group, 25% of patients presented late complications, among which we found mainly a leakage of the endoprosthesis and thrombosis of branches with ischaemia of the lower limbs, prosthesis migrations and rupture. Among the patients who underwent AAR, 12.8% had late complications, which mainly included hernia.

The SF-36 HRQoL questionnaire was administered to the 151 survivors at the time of the study (91 of the AAR and 60 of the EVAR). In Table 2, we show the results of the health scales. The highest scores were recorded on the scales for Body Pain, Social Function, Physical Function, Physical Role and Mental Health. The mean scores ranged from 81.7 in Body Pain to 59.2 in Emotional Role. In the Physical Role, Body Pain and Social Function scales, the percentage of participants who had extreme scores (0 or 100) was higher. All scales have an interval of 0 to 100, although the median exceeded the score of 50 in all scales, which indicates that the distribution of patients was concentrated in the high values of each scale; however, the percentage of patients with maximum scores only exceeded or equalled 59% in the Physical Role. On the other hand, there were many scales in which the percentage of patients with a minimum score was less than 5%.

Table 3 shows the results of the summary component PCS and MCS. The result obtained for the Health Transition item showed that 57.6% of participants reported being “more or less the same as a year ago”, 30.4% were “better than the previous year” and only 12% said they were “worse”.

Patients who underwent AAR showed a significantly higher mean score than the EVAR group in Physical Function (*p* = 0.001), Vitality (*p* = 0.003), General Health (*p* = 0.037), Social Function (*p* = 0.023) and Mental Health (*p* = 0.006) (Table 4). Table 5 shows the values obtained in the mean scores of the two summary measures: PCS and MCS. The patients who underwent AAR showed higher scores in both measures, although the differences were only statistically significant in the MCS (*p* = 0.026).

## 4. Discussion

AAA represents an important public health problem [2]. Despite scientific and technological advances in the treatment of AAA, the choice of repair by open surgery or endoprosthesis continues to be a matter of controversy. Postoperative mortality, considered mortality within 30 days after surgery, is the most important parameter for measuring the results [10]. However, the increase in life expectancy represents another dimension of the effect of treatment, which has a great impact on the physical and psychological wellbeing of the patient [11]. In this study, the effects on the two surgeries on HRQoL were compared using the SF-36 questionnaire [9].

The scores obtained in the SF-36 in our patients contained a minimal proportion of lost information, since the nonresponse to the questionnaire was null, and the response rate was 100% (100% of the patients who intended to participate were interviewed).

The best scores for the group of patients were obtained in the scales for Body Pain, Social Function, Physical Function, Physical Role and Mental Health. In the Physical Role, Body Pain and Social Function scales, we found the highest percentage of participants who responded with an extreme score, although the median score exceeded 50 in all scales. The percentage of patients with a maximum score that exceeded or equalled 59% was found in Physical Role, and in almost all the scales of the questionnaire, the percentage of patients with a minimum score was less than 5%. This indicates that in this group of patients, the SF-36 questionnaire allowed recording both the worsening of health (since the percentage of minimum scores is low and can grow) as well as improvements in health (the percentage of maximum score can grow on most scales).

In the scales of General Health, Vitality and Emotional Role, most patients obtained scores of approximately 50, and the lowest number of patients had scores at both ends of the scale. We relate these results to the fact that developing postoperative complications increases the probability of not reaching a high final score in these health domains, both for EVAR and AAR, interfering with physical health at work and in the activities of daily living. The results obtained in the two summary measures have a greater impact on the mental component of health more than the physical component.

For the total number of patients undergoing AAR, 7.9% assessed their health as much better compared to the previous year, 22.5% reported that they were somewhat better and 57.6% reported they were more or less the same. It is noteworthy that only 11.3% felt that their health was worse, and only 0.7% reported being much worse.

However, the HRQoL results were different according to the surgical technique used to repair AAA. When comparing the results of the SF-36 scales in AAR and EVAR patients, we obtained better scores in the scales of Physical Function, Vitality, General Health, Social Function and Mental Health in patients who underwent AAR than in those who underwent EVAR. This is statistically significant, mainly in the Social Function and Vitality scales. These results are related to the appearance of late complications in patients treated with EVAR. These patients manifested feelings of fatigue and exhaustion secondary to iterative consultations, both for new diagnoses, treatments of endoleaks, or symptoms and residual intermittent claudication in the lower limbs. These data correlated with those obtained in the literature, where we found that EVAR has a high rate of complications and reoperations [12,13]. The MCS scores were also better in the group of patients who underwent AAR.

We found that the greatest limitation of EVAR was the need for long-term follow-up using imaging techniques due to the high rate of late postoperative complications, which in our study group was 25%. Among the patients who underwent AAR, only 12.8% had late complications, mainly involving hernia. In a study conducted by Vallejo P. et al., on a sample of 64 patients who underwent EVAR, the same postoperative results were obtained [14].

The study of HRQoL reflects the burden of the disease from the point of view of the patient; therefore, they should be treated individually to meet their needs because their main concern is their symptoms regardless of whether the condition is serious. The results of the HRQoL that we obtained provide us with a comprehensive view of the patient and complement the traditional objectives of morbidity and mortality, documenting the benefits of the treatment and possible side effects from their subjective perception. The SF-36 questionnaire was developed for use in the *Medical Outcome Study* (MOS) from a wide range of questionnaires that included 40 concepts related to health through age, disease and treatment groups [15]. As a generic tool, the SF-36 questionnaire is appropriate for the evaluation of the results of an intervention aimed at prolonging life expectancy in a patient who may be asymptomatic with respect to the disease, as is the case for many AAA patients [16].

Although some studies have questioned the usefulness of the SF-36 questionnaire in older people [17], the high degree of comprehension of the questionnaire among our study participants and the high completion rate of all its items show that it is a useful instrument when administered by using personal interviews.

The health scales (Physical Function, Physical Role and Body Pain), which are correlated with the physical component, respond more to treatments that change physical morbidity, while the scales that collect the mental component (Mental Health, Emotional Role and Social Function) respond more to therapies that focus on mental health. Two of the scales (Vitality and General Health) are correlated with both components [18].

The content validity of the SF-36 has been compared with that of other widely used generic health surveys [19,20]. Systematic comparisons indicate that the SF-36 includes the eight most frequently measured health concepts; however, there are other health areas, such as sleep adequacy, cognitive functioning and sexual functioning, that are not included in the questionnaire. Being a generic measure, the symptoms and specific problems of a particular condition are not included; however, they are appropriate for evaluating an unexpected or expected result after an intervention. Elective AAA repair generally does not intend to improve patient symptoms, but it is expected that the extension of life expectancy will not decrease the HRQoL of a patient [16]. Another limitation of our study is that it reflects the experience of a single centre and that the collection of information from the patients was carried out in a cross-sectional manner, creating an opportunity to carry out a randomised study with longitudinal developments in the collection of information.

## 5. Conclusions

The conclusion reached is that patients who underwent AAR demonstrated better results on the HRQoL test than those who underwent EVAR. EVAR patients presented a very significant decrease in long-term HRQoL in the dimensions of Social Function and Vitality due to the greater number of late complications related to the endoprosthesis. The AAR patients presented a less significant decrease in HRQoL on the Emotional Role and Social Function scales, which we relate in this case to sexual impotence and the appearance of late herniations.

## Figures and Tables

**Table 1:** Characteristics and Complications of Patients Operated for AAA.

	AAR (*n* = 109)	EVAR (*n* = 69)	*p*-Value
AGE (mean ± SD)	71 (7)	76 (7)	0.09
Diabetes	19 (17.4%)	9 (13%)	0.49
Intermittent claudication	3 (2.7%)	1 (1.7%)	0.56
Lerich’s syndrome	1 (1%)	0	--
Ischemic heart disease	11 (10%)	11 (15.9%)	0.34
ACVA	2 (1.8%)	5 (7.2%)	0.25
EPOC	9 (9.6%)	8 (11.5%)	0.38
Chronic renal failure	16 (14.6%)	7 (10.1%)	0.27
Hyperuricemia	5 (4.5%)	0	--
Hypothyroidism	4 (3.6%)	2 (2.8%)	0.69
Prostate Pathology	16 (14.2%)	7 (10.1%)	0.47
SIZE OF THE ANEURISM			
40–56 mm	41 (44%)	30 (43.8%)	0.08
57–73 mm	28 (30%)	38 (55%)	
73–90 mm	10 (10.7%)	1 (1.6%)	
>90 mm	14 (15%)	0	
EARLY POSTOPERATIVE COMPLICATIONS			
Paralytic ileus	13 (11.9%)	0	--
Acute Renal Failure	11 (10%)	0	--
Respiratory distress	8 (7.3%)	0	--
Haemorrhagic shock	6 (5.5%)	1 (1.4%)	0.14
Bronchopneumonia	4 (3.6%)	0	--
Femoral pseudoaneurysm	0	1 (1.4%)	--
Paresis in LL	0	1 (1.4%)	--
Total (*)	30 (27.5%)	2 (2.8%)	0.001
LATE POSTOPERATIVE COMPLICATIONS			
Eventration	6(5.4%)	0	--
Ischemic Colitis	1(0.9%)	0	--
Peritonitis	1(0.9%)	0	--
Surgical wound dehiscence	1(0.9%)	0	--
Stent leak	0	7 (10.1%)	--
Iliac stenosis	0	2 (2.8%)	--
Lower limb thrombosis	0	3 (4.3%)	--
Acute lower limb ischemia	0	2 (2.8%)	--
Thrombophlebitis in Lower Limb	0	1 (1.4%)	--
Iliac pseudoaneurysm	0	1 (1.4%)	--
EVAR branch thrombosis	0	1 (1.4%)	--
Lower limb paraesthesia	0	1 (1.4%)	--
Total (*)	9 (8.2%)	18 (26%)	0.013
HOSPITAL STAY days (mean)	10 (7)	4.14 (4)	0.001

(*) Some patients presented more than one complication.

**Table 2:** Distribution of the scores of the Spanish version of the SF-36 health questionnaire for the total number of patients operated on for AAA.

	%N Answer	Cronbach’s Alpha	Correlation Person	Mean	SD	Percentile 5	Rank	Maximum	Minimum	Percentile 25	Percentile 75	Percentage with Maximum Score	Percentage with Minimum Score
Physical Function	0	0.88	0.88–0.54	76.2	22	80	100	100 (14)	0 (2)	70	95	9.3	1.3
Physical Role	0	0.88	0.78–0.54	74.3	37	100	100	100 (90)	0 (19)	50	100	59.6	13.9
Body Ache	0	0.75	0.60–0.89	81.7	23.5	90	100	100 (71)	0 (1)	70	100	47.7	0.7
General Health	0	0.70	0.82–0.56	60.8	18.7	65	85	100 (2)	15 (2)	45	75	1.3	1.3
Vitality	0	0.80	0.82–0.44	64.8	21.1	70	95	100 (4)	5 (1)	50	80	2.6	0.7
Social Function	0	0.86	0.93–0.76	80	25	87	100	100 (66)	0 (2)	75	100	43.7	1.3
Emotional Role	0	0.88	0.84–0.60	59.2	15.4	66	100	100 (1)	0 (2)	66	66	0.7	1.3
Mental Health	0	0.87	0.81–0.64	73.3	21.5	80	80	100 (17)	20 (6)	60	88	11.3	4.0

**Table 3:** Summary Scores of Total Patients in the Study Group.

(*n* = 151)	Physical Health	Mental Health
Mean	35.9582	34.9354
SD	10.24146	8.24213
Median	39.7917	36.2500
Minimum	4.79	8.50
Maximum	48.75	48.75

**Table 4:** Scores of the SF-36 Questionnaire in Patients Intervened Using AAR and EVAR.

		Physical Function	Role Physical	Body Ache	General Health	Vitality	Social Function	Role Emotional	Mental Health
AAR (*n* = 93)	Mean	80.05	74.46	82.47	63.01	67.90	81.99	58.42	75.48
	SD	21.23	37.76	22.58	20.82	22.20	25.76	16.04	23,46
	Median (Rank)	85 (100)	100 (100)	90 (100)	65 (85)	75 (100)	100 (100)	66.67 (100)	80 (80)
EVAR (*n* = 58)	Mean	69.05	71.55	80.52	56.47	58.62	75.65	59.77	68.83
	SD	22.48	38.74	24.95	14.23	18.77	25.15	14.98	18.21
	Median (Rank)	75 (100)	100 (100)	90 (100)	57.5 (55)	60 (80)	87.5 (100)	66.67 (67)	72 (80)
*p* value		0.001	0.623	0.704	0.037	0.003	0.023	0.562	0.006

**Table 5:** Summary measures in patients operated on for AAA by AAR and EVAR.

		Physical Health	Mental Health
AAR (*n* = 93)	Median	41.45	38.05
	IQR	43.46	40.25
EVAR (*n* = 58)	Median	38.48	34.06
	IQR	40.01	25.88
	*p* value (*)	0.032	0.003
Total (*n* = 151)	Median	39.79	36.25
	IQR	43.96	40.25

IQR: Interquartile range. (*) Mann–Whitney U test.

## Data Availability

The data used to support the findings of the present study are available from the corresponding author upon request.
